# Exploring anti-androgen therapies in hormone dependent prostate cancer and new therapeutic routes for castration resistant prostate cancer

**DOI:** 10.3389/fendo.2022.1006101

**Published:** 2022-10-03

**Authors:** Anna E. Harris, Veronika M. Metzler, Jennifer Lothion-Roy, Dhruvika Varun, Corinne L. Woodcock, Daisy B. Haigh, Chantelle Endeley, Maria Haque, Michael S. Toss, Mansour Alsaleem, Jenny L. Persson, Lorraine J. Gudas, Emad Rakha, Brian D. Robinson, Francesca Khani, Laura M. Martin, Jenna E. Moyer, Juliette Brownlie, Srinivasan Madhusudan, Cinzia Allegrucci, Victoria H. James, Catrin S. Rutland, Rupert G. Fray, Atara Ntekim, Simone de Brot, Nigel P. Mongan, Jennie N. Jeyapalan

**Affiliations:** ^1^ University of Nottingham Biodiscovery Institute, University of Nottingham, University Park, Nottingham, United Kingdom; ^2^ Department of Applied Medical Science, Applied College, Qassim University, Qassim, Saudi Arabia; ^3^ Department of Molecular Biology, Umeå University, Umeå, Sweden; ^4^ Department of Biomedical Sciences, Malmö Universitet, Malmö, Sweden; ^5^ Department of Pharmacology, Weill Cornell Medicine, New York, NY, United States; ^6^ Department of Urology, Weill Cornell Medicine, New York, NY, United States; ^7^ Englander Institute for Precision Medicine, Weill Cornell Medicine, New York, NY, United States; ^8^ School of Biosciences, University of Nottingham, Nottingham, United Kingdom; ^9^ Department of Oncology, University Hospital Ibadan, Ibadan, Nigeria; ^10^ Comparative Pathology Platform (COMPATH), Institute of Animal Pathology, University of Bern, Bern, Switzerland

**Keywords:** Therapy, anti-androgen, castration resistant prostate cancer, PARP inhibitors, epigenetic targeted treatment

## Abstract

Androgen deprivation therapies (ADTs) are important treatments which inhibit androgen-induced prostate cancer (PCa) progression by either preventing androgen biosynthesis (e.g. abiraterone) or by antagonizing androgen receptor (AR) function (e.g. bicalutamide, enzalutamide, darolutamide). A major limitation of current ADTs is they often remain effective for limited durations after which patients commonly progress to a lethal and incurable form of PCa, called castration-resistant prostate cancer (CRPC) where the AR continues to orchestrate pro-oncogenic signalling. Indeed, the increasing numbers of ADT-related treatment-emergent neuroendocrine-like prostate cancers (NePC), which lack AR and are thus insensitive to ADT, represents a major therapeutic challenge. There is therefore an urgent need to better understand the mechanisms of AR action in hormone dependent disease and the progression to CRPC, to enable the development of new approaches to prevent, reverse or delay ADT-resistance. Interestingly the AR regulates distinct transcriptional networks in hormone dependent and CRPC, and this appears to be related to the aberrant function of key AR-epigenetic coregulator enzymes including the lysine demethylase 1 (LSD1/KDM1A). In this review we summarize the current best status of anti-androgen clinical trials, the potential for novel combination therapies and we explore recent advances in the development of novel epigenetic targeted therapies that may be relevant to prevent or reverse disease progression in patients with advanced CRPC.

## Introduction

**Figure 1:**
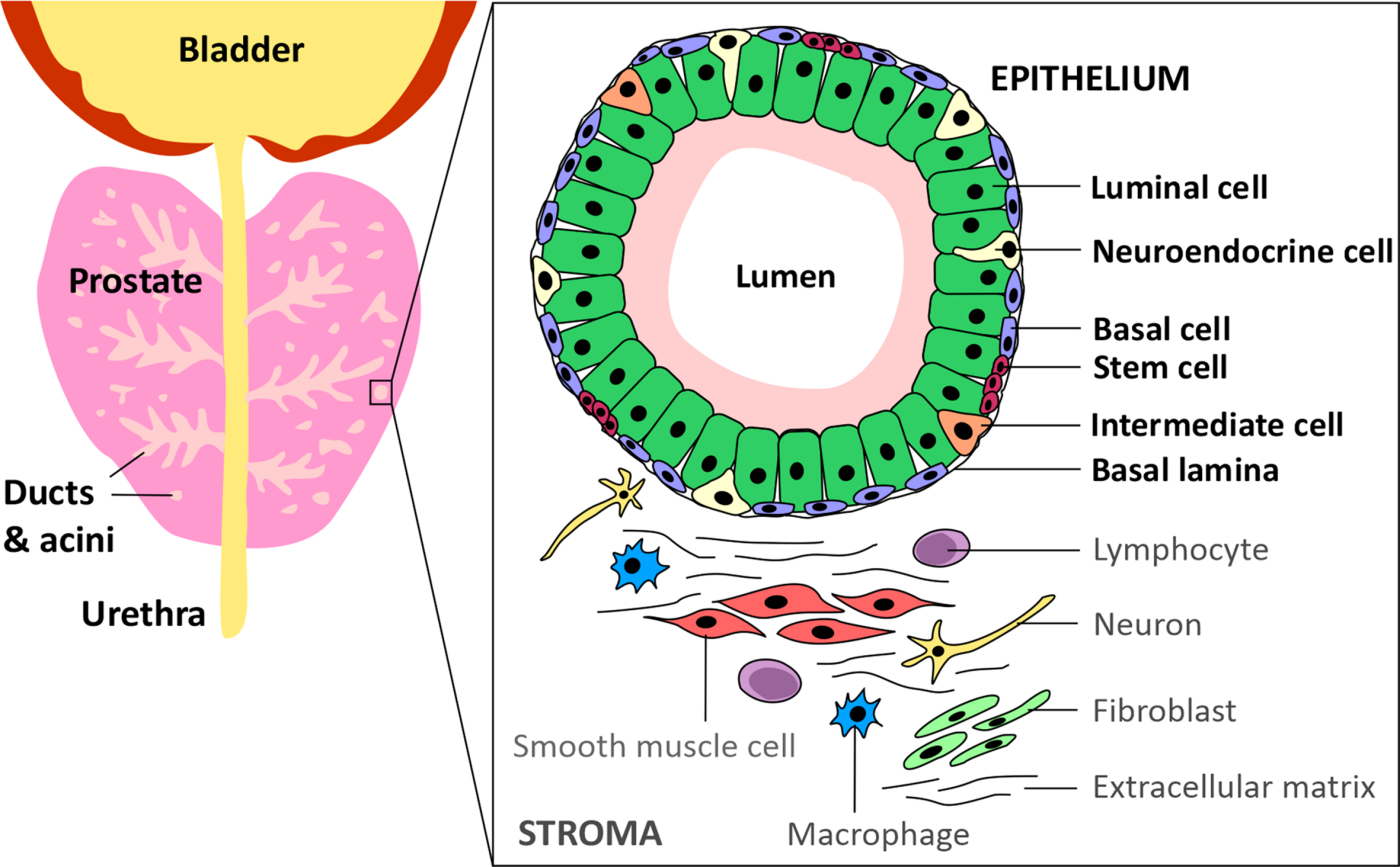
Schematic of the prostate epithelium and stroma. The prostate glandular structure is made up of ducts and acini. The epithelium that surrounds the lumen contains luminal, intermediate, basal, neuroendocrine and stem cells. The stroma contains extracellular matrix, immune cells, fibroblasts, neurons and smooth muscle cells.

Prostate cancer (PCa) is the most commonly diagnosed cancer in individuals with a prostate, with an estimated 268,490 new cases and 34,500 deaths predicted to occur in the USA alone over 2022 (1). Acinar adenocarcinoma is the most common type of all prostate cancers and accounts for around 95% of cases, whereas ductal adenocarcinoma accounts for only 0.4-0.8% (2). The epithelium of the ducts and acini is mainly composed of luminal and basal cells, but also intermediate, neuroendocrine and stem cells (**Figure 1**) (3). Primary PCa predominantly has a luminal cell-like phenotype with atypical glands, enhanced androgen signalling and absence of a continuous basal cell layer (4, 5). Luminal cells are terminally differentiated secretory cells of the prostate gland that are responsible for the exocrine activity of the prostate. They express high levels of Androgen receptor (AR) and secrete proteins including the prostate specific antigen (PSA) and prostate acid phosphatase (PacP) into the lumen of the gland (3). Basal cells, which express very little or no AR, are in direct contact with luminal cells *via* gap junctions and form a barrier between the luminal cells and the stroma and, like luminal cells, basal cells can give rise to cancers (4, 6). Prostate stem cells make up ~0.1-3% of the epithelial cell population and are believed to reside within “niches” near the basement membrane. The third most common cell type in the prostate are neuroendocrine cells which have neuronal characteristics (7–9). However, only about ~0.5-2% of prostate cancer patients have neuroendocrine prostate cancer (NePC) at initial diagnosis and NePC emergence often occurs after patients have received androgen deprivation therapies (ADTs), consequently NePC accounts for ~25% of all metastatic prostate cancers (10–12). NePC accounts for ~25% of all metastatic prostate cancers, is highly proliferative, aggressive and can lack AR expression, thus represents a major therapeutic challenge (12, 13). This review will discuss the androgen receptor signalling pathway, current treatments targeting androgen production and AR antagonists, moving forwards towards emerging treatment options for advanced castration resistant PCa (CRPC) and will consider how novel approaches to suppress androgen signalling may reduce or delay development of treatment-emergent NE-like PCa.

## Mechanisms of androgen receptor signaling

**Figure 2:**
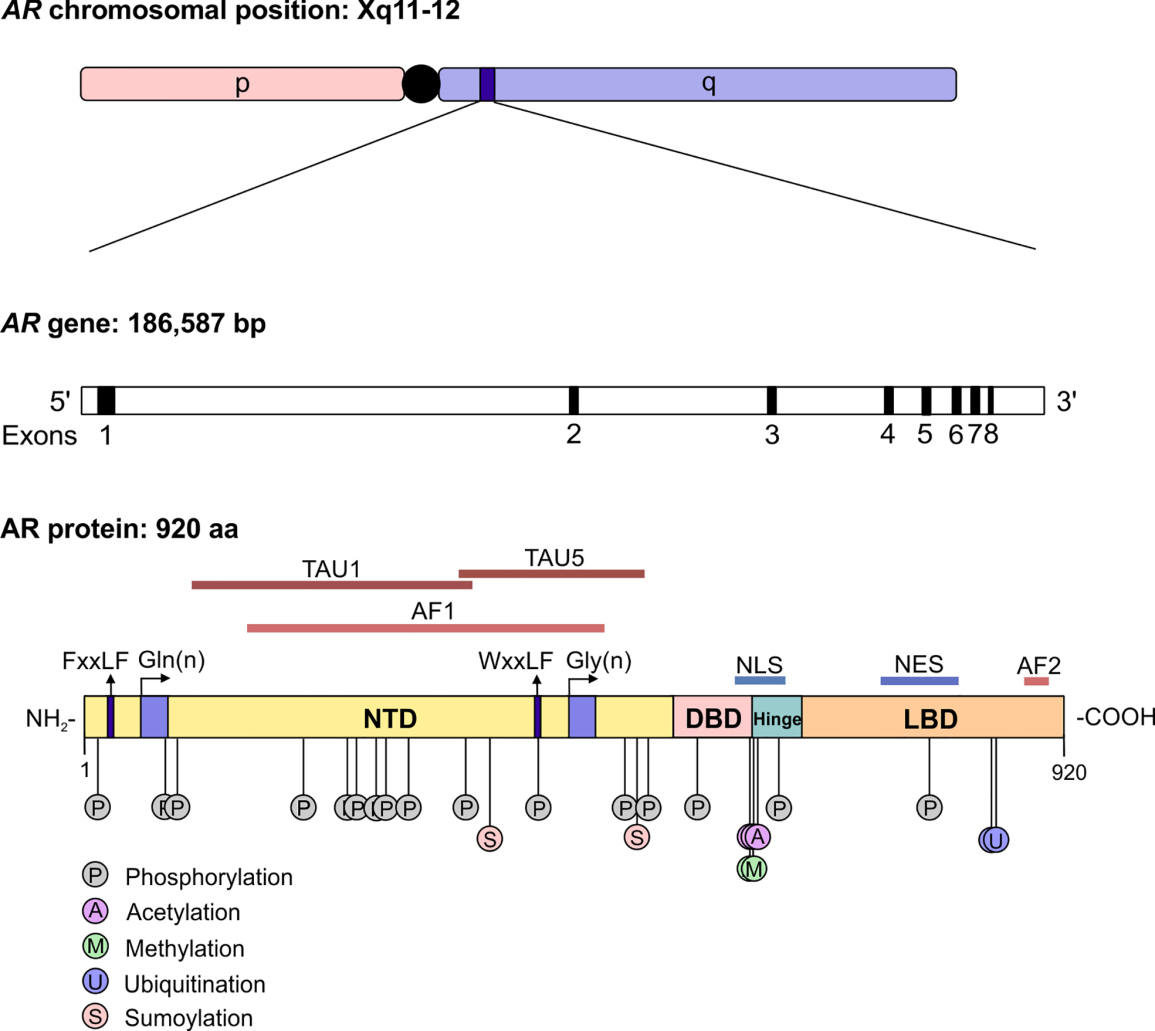
The structure of the androgen receptor gene and protein. The AR gene is situated on position q11-12 of chromosome X and contains 8 exons. The protein reference sequence NM_000044.3 is comprised of 920 amino acids and is composed of different domains which are depicted. In addition, posttranslational modifications known to influence AR function are shown. AR, androgen receptor; bp, base pair; NTD, N-terminal domain; DBD, DNA binding domain; LBD, ligand binding domain; AF, activation function; TAU, transcription activation unit; NLS, nuclear localisation signal; NES, nuclear export signal.

**Figure 3:**
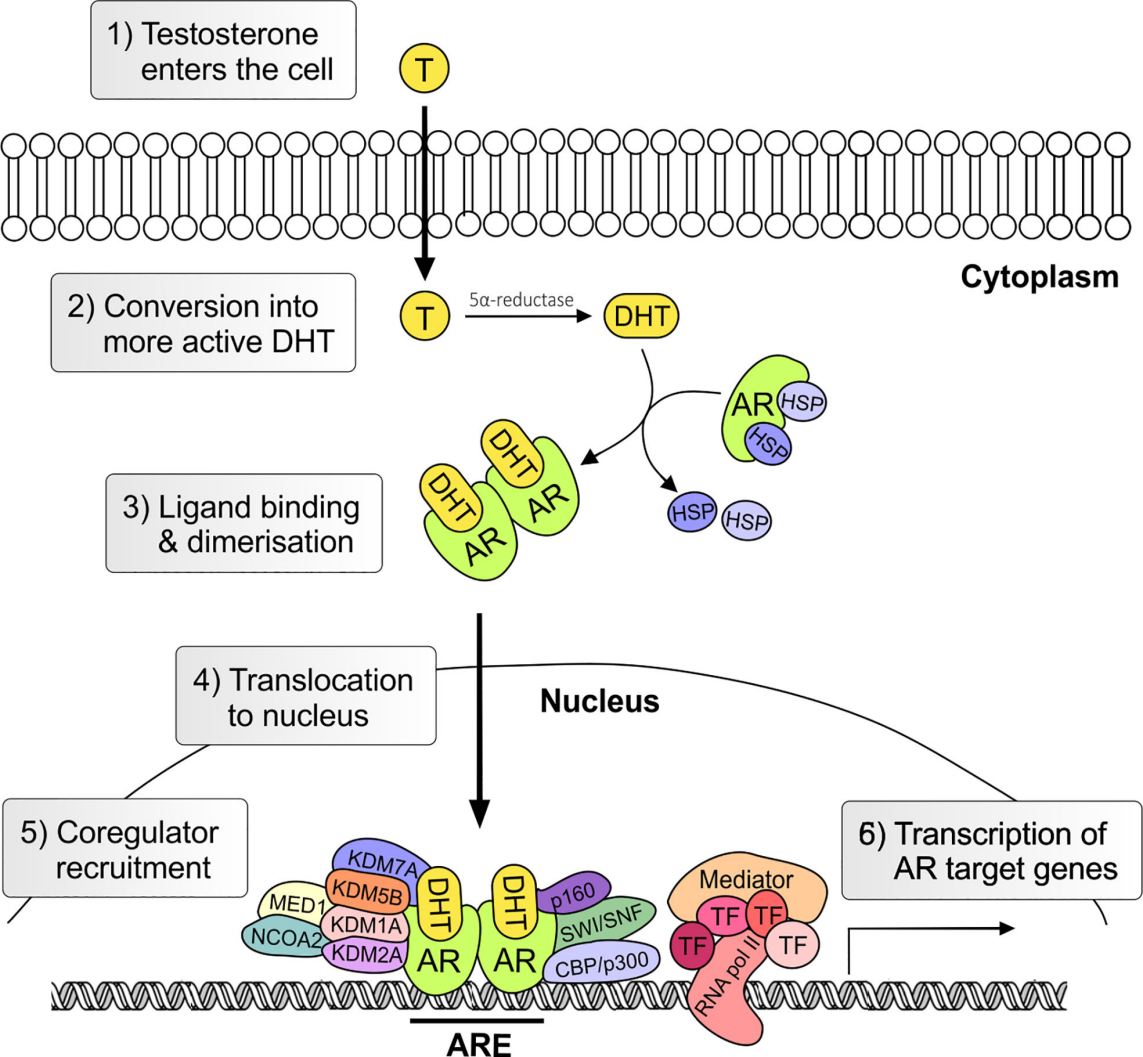
Genomic androgen receptor signaling pathway. Androgens, such as testosterone and dihydrotestosterone, enter the cell and are converted in the more active metabolite (5α-DHT) by the steroid-5α-reductase. Upon ligand binding heat stress protein (HSP) chaperones are released and AR undergoes conformational change and dimerization. In the nucleus the AR together with co-regulators activates the transcription of androgen regulated genes. T, testosterone; AR, androgen receptor; DHT, 5α-dihydrotestosterone; HSP, heat shock; TF, transcription factor; ARE, Androgen Response Element.

The androgen receptor (AR) is the primary mediator of androgen action. The main physiological androgens, testosterone and it more potent derivative, dihydrotestosterone (5-α-DHT), have long been recognized to play an important role in the initiation and progression of PCa (14). The transcriptional effects of androgens are mediated by the androgen receptor (AR), a member of the ligand dependent superfamily of transcription factors (15). The *AR* gene is located on chromosome X and spans ~186,587 base pairs (bp). The protein coding region is ~2757 nucleotides long and the protein is comprised of ~920 amino acids and possess polymorphic glutamine and glycine tracts (NM_000044.3, **Figure 2**). The 110 kDa AR protein is encoded by eight exons and contains four domains (i) the N-terminal domain (NTD), (ii) the DNA-binding domain (DBD), (iii) the hinge region and (iv) the ligand binding domain (LBD) (16). Within the COOH-terminus of the DBD and hinge region there is a so-called nuclear localization signal (NLS) which is responsible for the transport of the AR into the nucleus (17) (**Figure 2**). Agonist binding to the AR induces homodimerization, conformational changes and nuclear translocation (**Figure 3**). The AR homodimer usually is a “head-to-head” formation (18) and preferentially binds to the androgen response element (ARE) on the DNA (19, 20). The AR harbors two activation functions, the constitutively active activation function-1 (AF-1) is located in the NTD, whereas the ligand-dependent activation function-2 (AF-2) is situated in the LBD (16, 17). AR coregulator proteins bind to the hydrophobic pocket of AF-2 through their LxxLL (L = leucine, x = any amino acid) motif. This motif is highly conserved between nuclear receptor interacting proteins such as the steroid receptor coactivator-1 (SRC-1) and was shown to be necessary and sufficient to mediate coregulator binding to the LBD of the nuclear receptor (21). He et al. found a second motif, namely F*XX*LF, which is also present in the NTD of the AR (FQNLF) (22). This motif is therefore important for both interactions within AR itself and AR interaction with coregulator proteins. Coregulator proteins often share the same binding motif, however, the way the binding pocket of AR can bind coregulator proteins specifically is by electrostatic interactions with positively or negatively charged amino acids flanking the motif (23). The transcriptional activity of AR requires the recruitment of pioneer factors such as FoxA1 (24) and epigenetic co-regulator proteins (**Figure 3**), (25–30).

## Androgen deprivation therapies

**Figure 4:**
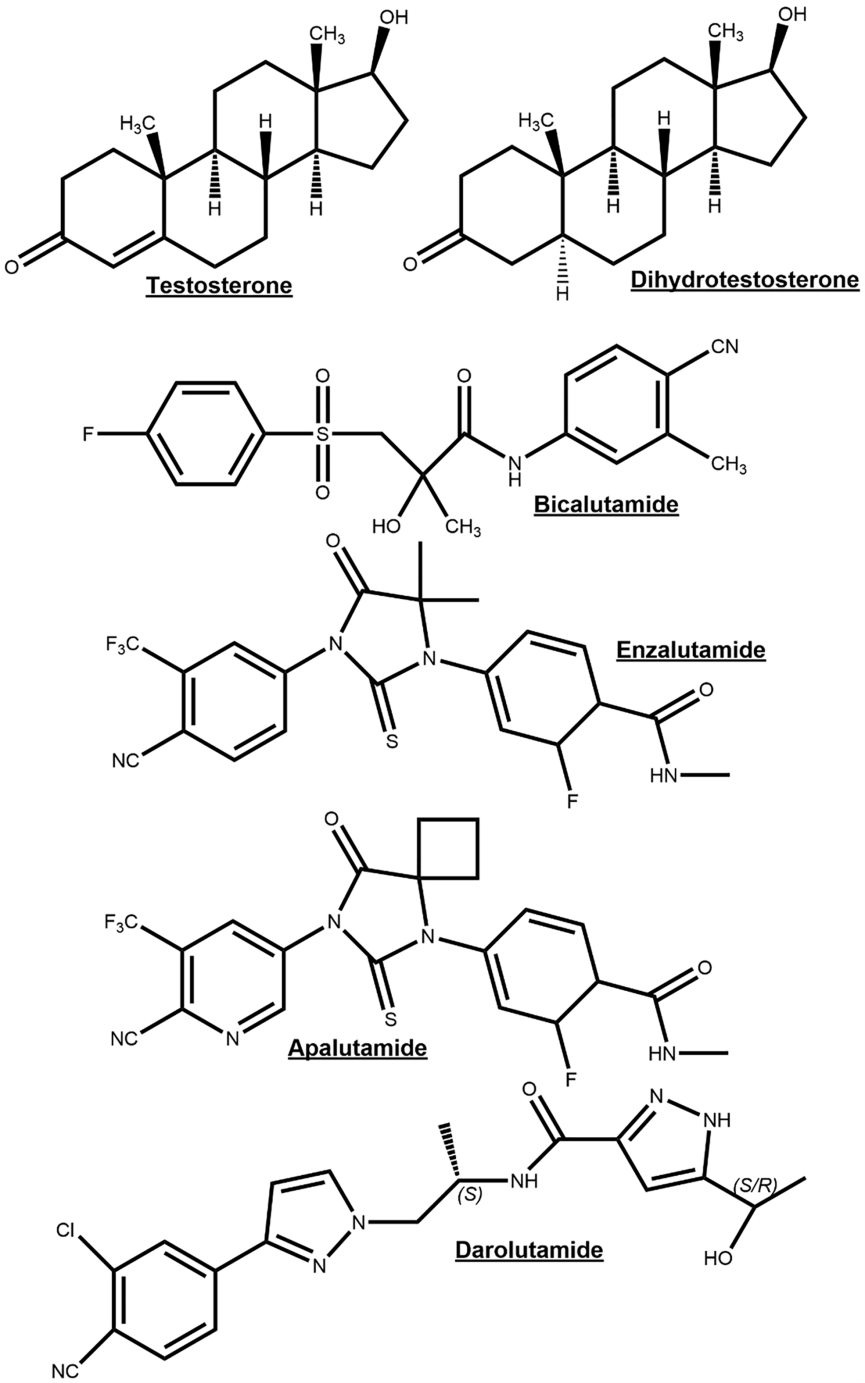
Chemical structures of the endogenous androgen receptor (AR) agonists testosterone and dihydrotestosterone for comparison to AR-antagonists bicalutamide, enzalutamide, apalutamide and darolutamide.

It has long been recognized that PCa initiation and progression is androgen-dependent and for this reason depriving PCa cells of androgen and androgen receptor function through inhibition of androgen biosynthesis or AR function will act to suppress tumorigenesis. Luteinizing hormone releasing hormone (LHRH) (also referred to as Gonadotropin Hormone-Releasing Hormone (GHRH)) agonists are androgen synthesis blockers which suppress androgen production by acting on the hypothalamus–pituitary–gonadal axis, whereby sustained stimulation of the LHRH receptor results in the downregulation of testosterone after an initial surge (31). LHRH antagonists bind directly to the GnRH receptor so act faster and without causing a testosterone surge and may be protective against neurodegenerative and cardiovascular disease compared to agonists (32–34). However, in LHRH agonists (e.g. Goserelin) and antagonist (e.g. Degarelix) treatments, androgen precursors released from the adrenal glands remain unaffected and can be metabolized into 5α-DHT, an AR agonist (35). Abiraterone irreversibly inhibits the Cytochrome P450 17 α-hydroxysteroid dehydrogenase (CYP17A) enzyme which is responsible for the conversion of pregnenolone to 17-OH Pregnenolone and a lyase activity to further convert 17-OH Pregnenolone into dehydroepiandrosterone (36). These are precursor molecules required for androgen biosynthesis in both testes and adrenal glands (36). Androgen synthesis blockers are often combined with androgen receptor antagonists, which compete with physiological androgens for AR binding sites. Flutamide was one of the first anti-androgens and has been shown to have positive effects on therapy response and PCa patient survival when combined with surgical or chemical castration (37). Bicalutamide is thought to have a more favorable tolerability profile relative to Flutamide (38). Later studies with advanced prostate cancer patients have shown that Bicalutamide combined with an LHRH agonist resulted in improved PSA levels and overall survival compared to LHRH agonist treatment alone (39, 40). The newer generation AR antagonists, Enzalutamide, Apalutamide, and Darolutamide, have a greater affinity for AR than Flutamide and Bicalutamide, and additionally block nuclear translocation of the AR, coactivator recruitment and DNA binding (41–43) (**Figure 4**). These newer generation of AR antagonists are discussed later in the review.

## Current and emerging therapies for CRPC

Despite recent success in developing more specific and effective ADTs, current ADTs are still only effective for ~24 months after which patients commonly progress to a lethal and incurable form of PCa, termed castration-resistant prostate cancer (CRPC) (44). Previously CRPC has also been referred to as hormone-refractory or androgen-independent PCa, but it has become clear that most CRPC cases remain influenced by androgen receptor signaling (45, 46). The mechanisms behind persistent AR signaling with concomitant ADT-resistance are the topic of many recent reviews and include AR overexpression, hypersensitivy to low androgen, increased androgen levels, *AR* gene mutations, drug antagonist-to-agonist switching, ligand promiscuity, bypassing the AR signaling pathway, AR splice variants, *AR* genomic structural rearrangements (*AR*-GSRs), and alterations in transcription factors and AR co-regulators (44, 47, 48). These mechanisms are thought to be the result of selective pressure of ADTs and provide the tumor with a selective advantage in the absence of androgen (49).

Several phase I and II studies demonstrated reductions in PSA (with varying success across cohorts) with abiraterone acetate treatment, often in conjunction with prednisone and subsequently is a standard of care treatment for metastatic castration resistant prostate cancer (mCRPC) (NCT00473512, NCT02217566, NCT01309672). These results also confirm that CRPC commonly remains hormone driven (50, 51). Additionally, clinical trials have concluded that the addition of abiraterone acetate and prednisone to conventional ADT significantly increased overall survival and radiographic progression-free survival in men with locally advanced and metastatic hormone naïve prostate cancer (HNPC) [NCT01715285, NCT00268476 (51, 52)]. Many trials remain ongoing including a Phase I trial to determine the feasibility of using PSA response and testosterone level to guide the treatment with ADT and/or abiraterone plus prednisone (NCT03511196), which if successful could present a more personalized approach and a minimalization of side effects. Abiraterone is currently used as a standard treatment for patients with metastatic CRPC (53). A screen for higher affinity, more affective non-steroidal antiandrogens identified Enzalutamide as the most promising candidate molecule (43). It is currently approved for the treatment of advanced CRPC after having demonstrated in clinical trials its effectiveness after resistance to traditional ADT has occurred. Several trials have shown a significant reduction in risk of progression or death compared to first generation antiandrogens (NCT02446405, NCT01664923) (54, 55). It is currently in phase II/III trials for both localized and metastatic hormone sensitive PCa as a component of first line ADT (NCT02446405, NCT02677896, NCT02446444, NCT03860987, NCT03809000) (54, 56). The combination of abiraterone with prednisone and enzalutamide is also in phase 2 trials for preoperative neoadjuvant treatment (NCT03860987). Another recent phase II trial supported the use of abiraterone with prednisone as a first-line therapy followed by enzalutamide as a second-line drug for better clinical outcomes for mCRPC as abiraterone was found to retain no second-line activity following enzalutamide [NCT02125357, (57)]. This may represent a method of prolonging sensitivity to hormonal therapy. Even though there are promising outcomes, Enzalutamide has been shown to promote NePC trans-differentiation (58).

Following Enzalutamide, another second-generation anti-androgen, Apalutamide, was developed. The addition of Apalutamide to conventional ADT was found to significantly improve patient outcome in non-metastatic CRPC (nmCRPC; NCT01946204) (59). In addition, initial results from the TITAN trial (NCT02489318) showed the addition of apalutamide to ADT significantly improved the primary endpoints of overall survival and radiographic progression-free survival in patients with metastatic castration-sensitive PCa (60, 61). Apalutamide is US FDA approved for use in both nmCRPC and metastatic castration sensitive PCa. Ongoing trials featuring apalutamide include for neoadjuvant ADT prior to radical prostatectomy for patients diagnosed with localized high-risk prostate cancer (NCT03767244) and as part of a combination therapy for patients with rising PSA after radiation therapy and ADT (NCT03777982) or radical prostatectomy (NCT03141671).

Another promising drug is darolutamide (ODM-201) which is a structurally distinct androgen receptor antagonist, which holds a significant advantage over other anti-androgens, as it shows negligible brain penetrance, does not increase serum testosterone levels in mice, reduces the risk of seizures and was found to inhibit AR variants (62). A recent study in men with nmCRPC (ARAMIS trial, phase III, NCT02200614) showed a significant improvement of metastasis-free survival in patients who received darolutamide (n = 955) compared to patients who received placebo (n = 554) (63). Darolutamide was approved in July 2019 by the US FDA for treatment of nmCRPC in combination with a GnRH analog or in patients who have had a bilateral orchiectomy. Currently it is in phase 3 trials for treatment in combination with an LHRH agonist/antagonist or orchiectomy with radiation therapy for localised prostate cancer at very high risk of recurrence (NCT04136353) and with docetaxel in patients with metastatic hormone sensitive prostate cancer (NCT02799602) (64). It is also now in a phase 2 trial as a monotherapy for hormone naïve PCa with limited metastases (NCT02972060).

Therapies such as Abiraterone and androgen receptor antagonists (Enzalutamide, Apalutamide, and Darolutamide) have been used to treat CRPC but are not curative (65). Efforts to target the AR NTD have generally been unsuccessful. For this reason there is an urgent need for new therapies targeting AR variants in CRPC, including potentially blocking AR variant expression (66). Another potential avenue for future therapeutics is targeted degradation of AR. PROTAC (Proteolysis targeting chimera) is a novel small molecule technology that targets a specific protein of interest for ubiquitination by E3 ubiquitin ligases and subsequently degradation. Several potent PROTAC degraders of AR have been developed including ARV-110 which is currently in phase 1/2 clinical trial for mCRPC (NCT03888612).

## Current combination therapies

Despite the challenges of resistance, there are benefits of hormonal therapies and there are many on-going clinical trials to identify optimal combinations of hormonal therapies with chemotherapy, radiotherapy, and immunotherapy.

Chemotherapy, commonly docetaxel or cabazitaxel, is often given only in late stages of prostate cancer disease to prolong the life of patients. Recently it has been shown Cabazitaxel plus prednisone use as a third line treatment after disease progression with docetaxel and abiraterone or enzalutamide, is significantly more effective than the hormonal therapy (NCT02485691) (67).

Radiotherapy is both given after neoadjuvant treatment to treat localized prostate cancers and at late-stage prostate cancer to target metastatic sites. The Phase III RADICALS trial (NCT00541047) found that precautionary radiotherapy after surgery, resulted in no significant difference in recurrence after 5 years compared with observation (68). This provides a strong case that radiotherapy should only be given if the cancer is found to have progressed. Stereotactic body radiation therapy (SBRT), also known as stereotactic ablative radiotherapy (SABR), is a method of delivering very high doses of radiation precisely to the tumor. It is emerging from phase 2 trials (NCT02680587 ORIOLE) as a treatment to target oligometastases that has low-toxicity and significantly increases progression free survival compared to observational arm (69). It has the potential to be an effective treatment that could delay ADT and thus maintain patient quality of life. Other trials remain ongoing (NCT04115007).

Another potential new therapeutic approach, recently reviewed by Loizzo et al., is targeting autophagy to sensitize prostate cancer to other drugs (70). Autophagy has a pro-tumorigenic role by promoting survival through enabling prostate cancer cells to meet metabolic demand and avoid apoptosis. Inhibitors of autophagy have been investigated in clinical trials, including hydroxychloroquine, which was shown in a phase 2 trial to have some reducing effect on PSA progression in localized PCa with PSA progression after local treatment (NCT00726596) (71).

Theranostics (or Theragnostics) is a novel therapeutic strategy in the move towards precision medicine to co-deliver therapeutic and imaging functions within a single agent. This includes delivering cytotoxic levels of radiation which are targeted at a cellular level to specific biomarkers. Prostate-specific membrane antigen (PSMA) presents a potential target for this approach, [reviewed by (72)]. It has shown promising results over Cabazitaxel in mCRPC in phase II trials (73). However, as not all PCa express PSMA, this method is not a universal treatment (74).

## Immunotherapies and DNA repair targeted therapies: Precision medicine in practice?

The identification of clinically relevant biomarkers and/or genotypes is critical for effective personalized treatment approaches to enable and inform prioritizing patients for immuno-and targeted therapies. While there is great interest in the development of immunotherapies for cancer, PCa is considered to have an immunosuppressive microenvironment, with few infiltrative lymphocytes. Recently the use of high dose-rate brachytherapy was able to alter this with increase in immune infiltrates (75). There are currently two FDA approved immunotherapies. Sipuleucel-T is a cell-based immunotherapy approach in which a patient’s own antigen presenting cells (APCs) are harvested and activated against prostatic acid phosphatase (PAP), a highly expressed antigen in most prostate cancer cells, then infused back into the patient. It was approved for the treatment of mCRPC following a series of successful phase III trials (76). Secondly, Pembrolizumab is a monoclonal antibody that targets the anti-programmed cell death protein 1 (PD-1) receptor has shown efficacy in several cancer types (NCT02578680, NCT02256436, NCT02362594) (77). A recent study identified a subset of super responders’ patients, of approximately 1 in 20 men with mCRPC that had an increase in life expectancy of two years (NCT02787005) (78). Several phase 2 and 3 trials featuring Pembrolizumab are ongoing including a Phase 2 combining ADT, SBRT and pembrolizumab with or without intra-tumoral SD-101, a synthetic CpG oligonucleotide that enhances immune response, in patients with newly diagnosed hormone-naive oligometastatic prostate cancer (NCT03007732). Immunotherapies are generally well tolerated and there are many ongoing trials for the treatment of mPCa and localised PCa alongside radiotherapy. Identification of biomarkers which identify individuals likely to benefit from immunotherapies will enable more personalized use of such treatments.

Recent advances in biomarker discovery have enabled the more precise use of targeted therapies in PCa. A new class of anticancer drugs called PARP inhibitors (PARPi) have been developed and trialled successfully on several types of cancers including PCa. Poly (ADP-ribose) polymerases 1 and 2 (PARP1 and 2) play a crucial role in DNA damage repair (DDR) by sensing DNA damage, binding to the damaged DNA and recruiting DNA repair proteins to the damage site, through a process called PARylation (79). PARP inhibitors bind to the catalytic site of PARP1 and trap the enzyme at the location of DNA damage. This blocks the replication fork and eventually causes double strand breaks (DSB) in the DNA. Since the catalytic site is also responsible for PARylation, blocking of this site inhibits the recruitment of DNA repair factors, inhibiting PARP-mediated DDR (80).

A clinical trial in 2015 (NCT01682772) tested the efficacy of olaparib, a PARP inhibitor, in treating mCRPC and found that treatment with olaparib prolonged radiographic progression–free survival (rPFS) and reduced circulating tumor-cell counts. Importantly, a better response was observed in patients with tumors that had aberrations in DNA repair genes (81). An alternative PARP inhibitor, veliparib, was tested for its efficacy with abiraterone acetate and prednisone therapy (AAP) in a phase 2 clinical trial (NCT01576172) (82). Although no significant benefits were observed with veliparib in addition to AAP in mCRPC, the study reported better treatment outcomes with AAP in tumors where DNA repair gene defects were found on metastatic tumor tissue sequencing as compared to tumours without these defects (82).

Indeed, the success of PARP inhibition as a treatment strategy has been linked to the genetic context of the tumor and more specifically to the functionality of certain genes involved in DDR. PARP inhibition in breast and ovarian cancers with BRCA1/2 mutation represents the first clinical application of *synthetic lethality;* a term used to describe cell death following the genetic or pharmacological inhibition of a cellular pathway in the context of a distinct loss of function mutation in a separate pathway (where neither the primary mutation nor the treatment alone would be lethal to the cell) (83). BRCA 1 and 2 are involved in the resolution of DSBs *via* the homologous recombination repair (HRR) pathway. Where homologous repair of DSBs cannot be carried out, cells are forced to use more error-prone non-homologous end joining (NHEJ) which results in chromosomal instability and increased sensitivity to PARPi (83). BRCA germline mutations are present in a subset of patients with PCa and identifying these patients would allow the use of PARP inhibitors (84). Additionally, BRCA2 mutation has been identified in 2.5-5.3% of aggressive prostate cancers (85, 86) and the standardized incidence ratio of prostate cancer in males known to carry a pathogenic BRCA2 mutation was found to be 4.45 (87). Overall, mutations in DDR have been identified in 11-30% of prostate cancers and represent possible targets for synthetically lethal treatment approaches (86, 88, 89).

The profound trial (NCT02987543) aimed to compare olaparib against enzalutamide or abiraterone for the treatment of mCRPC with HRR gene alterations (79). It found that olaparib improved rPFS and objective response rate in comparison to treatment with enzalutamide or abiraterone, although concluded that further studies are required to better define the genetic mutations that will sensitize a cancer to olaparib. For patients with PCa and at least one alteration in BRCA1, BRCA2 or ATM (a serine threonine kinase involved in the processing of DSBs and in HRR) (90, 91), overall survival was significantly longer for patients that received olaparib (79). However, analysis from the TRITON2 study of PARP inhibition with rucaparib found that of the 49 patients with mCRPC identified to have ATM mutation, only 4.1% showed a significant decline in PSA following treatment (92). Neeb et al. identified ATM loss in 11% of a cohort of 631 patients with advanced prostate cancer and found that ATM knockout in a human prostate carcinoma cell line was associated with genetic instability. HRR was found to be reduced but not absent in these cells and response to rucaparib was inconstant between clones of the same cell line. Chemical inhibition of ATR (a kinase involved in DNA repair *via* cell cycle checkpoint control) was found to inhibit HRR in the ATM knockout cells and although cytotoxic effect was modest in the context of ATM knockout alone, addition of PARP inhibition significantly increased cell death. The efficacy of inhibiting both PARP and ATR was then confirmed in a xenographic model of a patient derived mCRPC cell line with loss of ATM (93). This study identifies the possible need for the HRR pathway to be completely inhibited in order for olaparib treatment to be most successful. ATM inhibition represents another potential application of precision medicine and a phase 2 clinical trial of combined treatment with olaparib alongside ATR inhibition in mCRPC is currently ongoing (NCT03787680). Other possible DDR genes of interest in PCa have been identified and include PALB2, FANCA, BRIP1 and RAD51 (92).

So far, olaparib has been approved by the FDA for use in mCRPC with identified mutation in HRR and rucaparib has been approved for mCRPC with BRCA mutation. Ongoing trials of PARP inhibition in prostate cancer and completed trials awaiting results are included in **Table 1**. Current evidence from both pre-clinical and clinical trials indicates that tumours with DNA repair gene deficiency often respond better to PARP inhibitors and other DDR-targeted therapies, due to possible synthetically lethal interactions. This paves a new direction for the use of precision medicine in treating PCa.

**Table 1:** Ongoing androgen deprivation and targeted therapies and clinical trials in prostate cancer– ordered by drug name.

Identifier	Treatment/Intervention	Phase	Trial Outcome
NCT00473512	Abiraterone acetate, Dexamethasone	1&2	C
NCT02125357	Abiraterone acetate, Enzalutamide	2	C
NCT01309672	Abiraterone acetate, Prednisone	2	PC, O
NCT02217566	Abiraterone acetate, Prednisone, Androgen deprivation therapy (ADT)	2	C
NCT01715285	Abiraterone acetate, Prednisone, Androgen deprivation therapy (ADT), Prednisone	3	C
NCT03012321	Abiraterone/prednisone, olaparib or Abiraterone/prednisone + Olaparib	2	O
NCT01085422	ABT-888 (oral PARP inhibitor), temozolomide	1	C
NCT03511196	Adaptive Androgen Deprivation Therapy (ADT), Abiraterone, Prednisone	1	O
NCT02489318	Apalutamide, Androgen Deprivation Therapy (ADT)	3	PC, O
NCT03767244	Apalutamide, Androgen Deprivation Therapy (ADT)	3	O
NCT01946204	Apalutamide, current treatment	3	PC, O
NCT03888612	ARV-110	1&2	O
NCT00541047	Bicalutamide, goserelin acetate, leuprolide acetate, radiation therapy	3	O
NCT02485691	Cabazitaxel, enzalutamide, abiraterone acetate, prednisone	4	C
NCT02893917	Cediranib + Olaparib vs Olaparib	2	O
NCT00268476	Celecoxib, Docetaxel, Prednisolone, ADT, Zoledronic Acid, Abiraterone,: Radiotherapy to the prostate, Enzalutamide, Metformin, Transdermal Oestradiol	2&3	O
NCT02200614	Darolutamide	3	C
NCT02972060	Darolutamide (ODM-201), ADT	2	O
NCT02799602	Darolutamide (ODM-201), Standard ADT, Docetaxel	3	O
NCT04136353	Darolutamide, Luteinizing Hormone-Releasing Hormone Analog, External Beam Radiotherapy	3	O
NCT01212991	Enzalutamide	3	C
NCT02677896	Enzalutamide	3	PC, O
NCT01664923	Enzalutamide, Bicalutamide	2	C
NCT02446444	Enzalutamide, Conventional NSAA, LHRHA, External Beam Radiotherapy (78 Gy in 39 fractions or 46 Gy in 23 fractions plus brachytherapy boost)	3	O
NCT02446405	Enzalutamide, NSAA, LHRHA or Surgical Castration	3	C
NCT03141671	GnRH, Bicalutamide, Salvage radiation, Abiraterone, Prednisone, Apalutamide	2	O
NCT03860987	Goserelin, Enzalutamide, Abiraterone, Prednisone, Radical Prostatectomy	2	O
NCT00726596	Hydroxychloroquine	2	C
NCT02854436	Niraparib	2	O
NCT04592237	Niraparib, cabazitaxel, carboplatin,cetrelimab,	2	O
NCT04194554	Niraparib, leuprolide, abiraterone acetate, Stereotactic body radiotherapy (SBRT)	1&2	O
NCT04030559	Niraparib, niraparib tosylate monohydrate	2	O
NCT04951492	Olaparib	2	O
NCT01682772	Olaparib	2	C
NCT03047135	Olaparib following radical prostatectomy	2	O
NCT01972217	Olaparib, abiraterone	2	PC, O
NCT05167175	Olaparib, abiraterone acetate, prednisone	2	O
NCT03787680	Olaparib, AZD6738 (ATR inhibitor)	2	O
NCT02324998	Olaparib, degarelix	1	C
NCT04336943	Olaparib, durvalumab	2	O
NCT03810105	Olaparib, durvalumab	2	O
NCT02987543	Olaparib, enzalutamide, abiraterone acetate	3	PC, O
NCT03317392	Olaparib, radium Ra 223 dichloride	1&2	O
NCT03516812	Olaparib, testosterone enanthane, testosterone cypionate	2	O
NCT05327621	Pamiparib	2	O
NCT02362594	Pembrolizumab	3	PC, O
NCT03834519	Pembrolizumab (MK-3475) and olaparib vs abiraterone or enzalutamide	3	O
NCT02578680	Pembrolizumab, Cisplatin, Carboplatin, Pemetrexed, Dexamethasone	3	PC, O
NCT02787005	Pembrolizumab, Enzalutamide	2	C
NCT02256436	Pembrolizumab, paclitaxel, vinflunine, docetaxel	3	C
NCT03007732	Pembrolizumab, SD-101, Leuprolide acetate, Abiraterone Acetate, Prednisone, Stereotactic Body Radiation Therapy	2	O
NCT03777982	Prednisone, Apalutamide, Abiraterone Acetate, LHRH Agonist or Antagonist	3	O
NCT03809000	Radiation Therapy, Enzalutamide, Bicalutamide, GnRH analog	2	O
NCT02952534	Rucaparib	2	C
NCT03533946	Rucaparib	2	O
NCT02975934	Rucaparib vs abiraterone acetate or enzalutamide or docetaxel	3	O
NCT04455750	Rucaparib, enzalutamide	3	O
NCT04179396	Rucaparib, enzalutamide, abiraterone	1	O
NCT02680587	SBRT	2	O
NCT04115007	Stereotactic Body Radiotherapy (SBRT), Standard of care	3	O
NCT04550494	Talazoparib	2	O
NCT04703920	Talazoparib, belinostat	1	O
NCT04734730	Talazoparib, bicalutamide, degarelix, goserelinacetate, leuprolide acetate, prednisone	2	O
NCT04821622	Talazoparib, enzalutamide	3	O
NCT04824937	Telaglenastat, talazoparib	2	O
NCT01576172	Veliparib, abiraterone acetate, prednisone	2	C

PC, primary completion; C, completed; O, on-going.

## Emerging epigenetic therapies for advanced prostate cancer

In recent years the focus on understanding cancer progression has led to several multicenter studies being able to stratify prostate cancer. This stratification has also highlighted the crucial role of epigenetic factors in cancer progression (94–100). Here we introduce the epigenetic factors that have been well- studied in prostate cancer and their potential therapeutic role as future treatment options.

**Figure 5:**
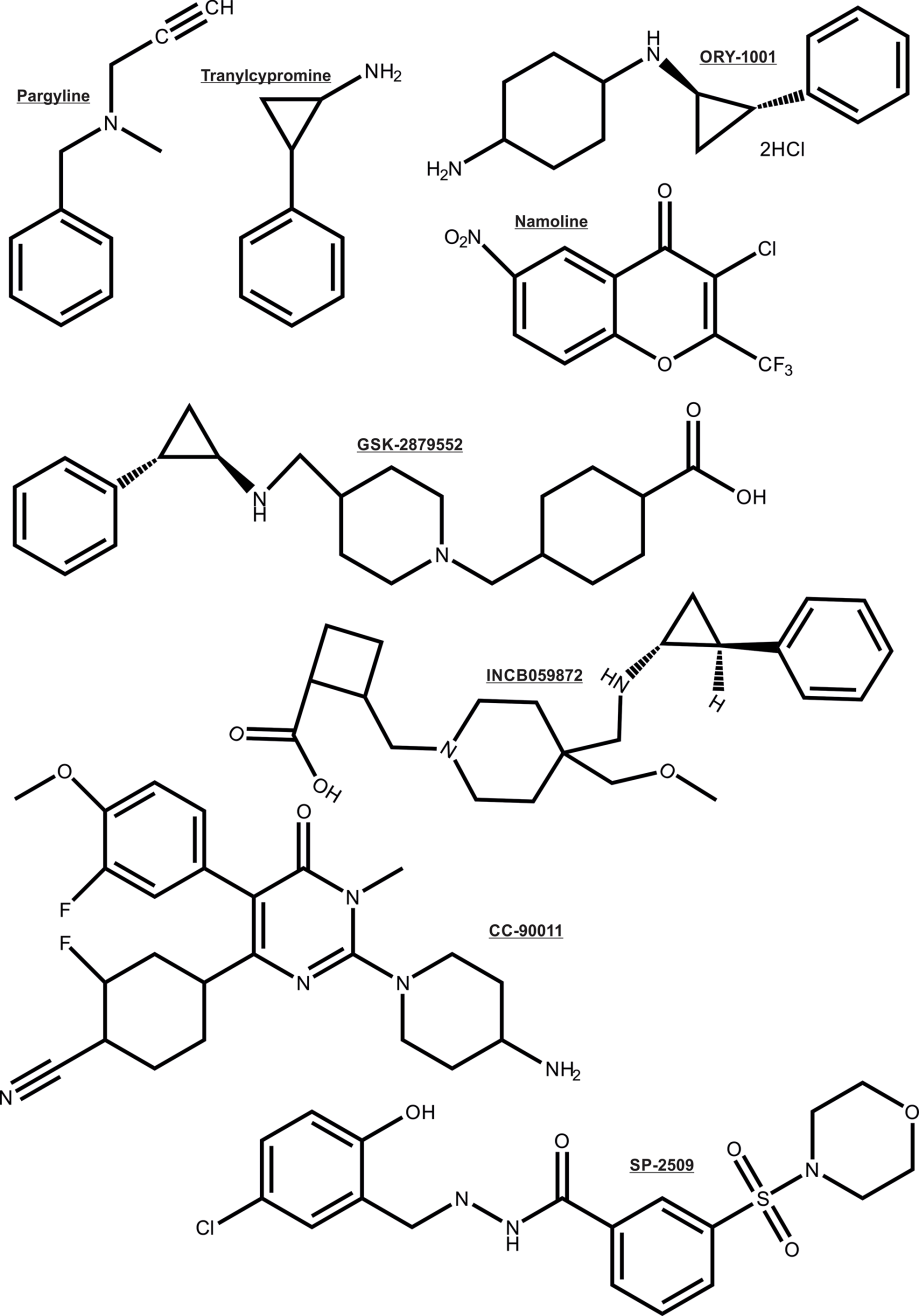
Chemical structure of the KDM1A inhibitors.

As noted earlier, the AR depends on multiple enzymatic distinct epigenetic coregulators, many of which are independently implicated in PCa. For this reason the focus is now shifting to the chromatin modifying coregulator proteins such as lysine demethylases (KDMs) that are key cofactors for cancer drivers such as AR (26, 27, 101–104). For these reasons, the recent entry of KDM1A pharmaco-inhibitors into cancer clinical trials herald a new step in the translation of innovative epigenetic therapies to the clinic (105), even though some of the trials were terminated due to no beneficial effects in the cancer types treated. Some KDM1A inhibitors being investigated in clinical trials to treat cancers are: ORY-1001, ORY-2001, GSK-2879552, IMG7289, INCB059872 and CC-90011 (chemical structure shown in **Figure 5**) (106). Other histone demethylases like KDM3A, KDM4B/KDM4C, KDM6A/KDM6B have also been associated with regulating the transcriptional activity of AR (107–110). Moreover, inhibitors simultaneously targeting KDM4 and KDM1 family enzymes have been developed and shown to be effective in inducing apoptosis of PCa and colon cancer cell lines (111). Thus, these KDMs form interesting epigenetic therapeutic targets in PCa.

Another important epigenetic factor in PCa is BRD4, a member of BET (bromodomain and extra terminal domain) subfamily. It binds acetylated histone lysine residues and has been shown to be important for recruiting RNA polymerase II to facilitate transcription. In CRPC, BRD4 interacts with AR and inhibition of BRD4 prevents AR from binding to its target genes, disrupting AR transcriptional activity [nicely reviewed by Pawar et al.; (112)]. Thus, BRD4 is being investigated as a potential therapeutic target in PCa. JQ1 and AZD5153 are BET inhibitors that are effective at selectively inhibiting BRD4 and have been shown to reduce proliferation and survival of PCa cells (113). It has been shown that mutations in *SPOP* gene in PCa often impart resistance to BET inhibitors (114). However, a molecule called PROTAC-BETd (ZBC260) has been shown to re-sensitize the resistant cells to BET inhibitors. Hence, it has been suggested that ZBC260 and BET inhibitor combination therapy may yield better treatment outcomes in cancers (115). BRD4 works together with E2F1 transcription factor, the activation of which induces lineage plasticity in treatment-emergent neuroendocrine PCa (t-NEPC). BET inhibitors have been reported to block this E2F1/BRD4 dependent mechanism and reduce tumor growth in t-NEPC (116). BRD4 also plays a role in transcriptional regulation of KDM5C, which is often overexpressed in CRPC. Knockdown of KDM5C tends to sensitize CRPC cells to BET inhibitors, suggesting an interplay between these proteins and uncovering a new pathway that could be targeted to develop treatments for CRPC (117). Recently, JQ1 and a KDM1A inhibitor, SP-2509, were tested together in CRPC and showed a synergistic effect at inhibiting growth of AR positive CRPC cells. Although, the sensitivity to these inhibitors was lost on AR knockdown (118).

EZH2 (enhancer of zeste homolog 2) is a methyltransferase that typically functions as part of the polycomb repressor complex 2 (PRC2). However, recently it was shown that it also exhibits a separate activation function when it binds to the promoter region of AR and plays a role in AR signaling. It also acts as a co-activator of AR in CRPC [reviewed by Jones et al. (110)]. Recently, a study found that inhibition of EZH2 enhanced enzalutamide activity and could potential overcome enzalutamide resistance in CRPC. This was thought to be due to inhibition of PSA transcription by EZH2 directly binding to PSA promoter region (119). Furthermore, EZH2 and EED (embryonic ectoderm development) can modulate AR expression and AR signaling pathways. A drug called astemizole disturbs EZH2-EED interaction and inhibits both, EZH2 and AR expression. Astemizole has been reported to promote autophagy in PCa cells and reduce tumor growth in castration-resistant mouse xenograft model with implanted VCaP cells (120). It has also been recognised that EZH2 is a major player in the evolution of NEPC-CRPC (121). A study recently showed how understanding the ECM components within the tumor environment can be utilized in organoid models and identified ECM-dependent EZH2 inhibition was able to sensitize NEPC to treatment (122). This has also brought to the forefront the regulation of EZH2 in PCa. The methylation and subsequent degradation of EZH2 by the methyltransferase SETD2, was recently shown to block metastasis in PCa, further suggesting that EZH2 inhibition in PCa could be of therapeutic advantage (123).

These studies provide hope for the development of combined epigenetic therapies in the future that effectively target two or more epigenetic factors significant in PCa.

## Conclusion

In conclusion, therapeutic strategies targeting the AR function remain the cornerstone for treating men with advanced PCa. However, the emergence of castrate resistant PCa and indeed treatment emergent neuroendocrine-like PCa, highlights the need for the development of newer more targeted pharmacological approaches to suppress pro-proliferative androgen signaling. With the emerging role of DNA repair and epigenetic factors in mCRPC and NEPC, identifying and repurposing drugs for these new factors in PCa could lead to better precision treatment options for individuals with advanced PCa.

The development of epigenetic targeting therapies represents a novel approach to suppress androgen signaling and could be an additional line of therapy after resistance has occurred to other approaches. Indeed, a precision medicine approach could also be applied to identify and treat individuals that would respond to immunotherapies, including methods of enhancing immune response and immune infiltration for effective immunotherapy. The numerous ongoing clinical trials will continue to identify the most effective combinations of hormonal therapies with chemotherapy, radiotherapy, immunotherapy and other novel therapies to improve both survival and quality of life of prostate cancer patients.

## Author contributions

AH, AN, and NM contributed to conception and design of the study. AH, VM, JL-R, DV, NM, and JJ review preparation. AH, VM, DV, NM, CR, AN, SB, and JJ wrote first draft. AH, DV, CW, CE, MH, MT, MA, JB, SM, AN, SB, and JJ wrote sections of the review. JP, LG, ER, BR, FK, LM, JM, JB, SM, CA, VJ, CR, RF, AN, SB and NM critical revision of review. All authors contributed to the article and approved the submitted version.

## Funding

We gratefully acknowledge the financial support of the University of Nottingham, the BBSRC; Doctoral training program (BB/M008770/1: AEH, VMM, DBH, CLW, JL-R, CSR, NPM) and (BB/T008369/1: DV, CE, MH) and Prostate Cancer UK RIA15-ST2-005 (NPM) and Prostate Cancer Foundation & John Black Charitable Foundation (20CHAL06: NPM).

## Conflict of interest

The authors declare that the research was conducted in the absence of any commercial or financial relationships that could be construed as a potential conflict of interest.

## Publisher’s note

All claims expressed in this article are solely those of the authors and do not necessarily represent those of their affiliated organizations, or those of the publisher, the editors and the reviewers. Any product that may be evaluated in this article, or claim that may be made by its manufacturer, is not guaranteed or endorsed by the publisher.
